# Distribution of Bexsero® Antigen Sequence Types (BASTs) in invasive meningococcal disease isolates: Implications for immunisation

**DOI:** 10.1016/j.vaccine.2016.08.015

**Published:** 2016-09-07

**Authors:** Carina Brehony, Charlene M.C. Rodrigues, Ray Borrow, Andrew Smith, Robert Cunney, E. Richard Moxon, Martin C.J. Maiden

**Affiliations:** a Department of Zoology, University of Oxford, South Parks Road, Oxford, United Kingdom; b Public Health England, Meningococcal Reference Unit, Manchester Royal Infirmary, Manchester, United Kingdom; c Scottish Haemophilus, Legionella, Meningococcus and Pneumococcus Reference Laboratory, Glasgow Royal Infirmary, Glasgow, United Kingdom; d College of Medical, Veterinary & Life Sciences, University of Glasgow, Glasgow, United Kingdom; e Irish Meningitis and Meningococcal Reference Laboratory, Temple Street Children’s University Hospital, Dublin, Ireland; f Department of Paediatrics, University of Oxford, John Radcliffe Hospital, Oxford, United Kingdom

**Keywords:** *Neisseria meningitidis*, Vaccine design, Molecular epidemiology, Genome, Surveillance, Bexsero® Antigen Sequence Type, BAST, Bexsero® Antigen Sequence Type, cc, clonal complex, fHbp, factor-H binding protein, IMD, invasive meningococcal disease, MATS, Meningococcal Antigen Typing System, MLST, multilocus sequence type, MRF-MGL, Meningitis Research Foundation Meningococcus Genome Library, NadA, neisserial adhesion A, NHBA, neisserial heparin-binding antigen

## Abstract

Serogroup B is the only major disease-associated capsular group of *Neisseria meningitidis* for which no protein-polysaccharide conjugate vaccine is available. This has led to the development of multi-component protein-based vaccines that target serogroup B invasive meningococcal disease (IMD), including Bexsero®, which was implemented for UK infants in 2015, and Trumenba®. Given the diversity of meningococcal protein antigens, post-implementation surveillance of IMD isolates, including characterisation of vaccine antigens, is essential for assessing the effectiveness of such vaccines. Whole genome sequencing (WGS), as realised in the Meningitis Research Foundation Meningococcus Genome Library (MRF-MGL), provides a rapid, comprehensive, and cost-effective approach to this. To facilitate the surveillance of the antigen targets included in Bexsero® (fHbp, PorA, NHBA and NadA) for protective immunity, a Bexsero® Antigen Sequence Type (BAST) scheme, based on deduced peptide sequence variants, was implemented in the PubMLST.org/neisseria database, which includes the MRF-MGL and other isolate collections. This scheme enabled the characterisation of vaccine antigen variants and here the invasive meningococci isolated in Great Britain and Ireland in the epidemiological years 2010/11 to 2013/14 are analysed. Many unique BASTs (647) were present, but nine of these accounted for 39% (775/1966) of isolates, with some temporal and geographic differences in BAST distribution. BASTs were strongly associated with other characteristics, such as serogroup and clonal complex (cc), and a significant increase in BAST-2 was associated with increased prevalence of serogroup W clonal complex 11 meningococci. Potential coverage was assessed by the examination of the antigen peptide sequences present in the vaccine and epidemiological dataset. There were 22.8–30.8% exact peptide matches to Bexsero® components and predicted coverage of 66.1%, based on genotype-phenotype modelling for 63.7% of serogroup B isolates from 2010/14 in UK and Ireland. While there are many caveats to this estimate, it lies within the range of other published estimates.

## Highlights

• Surveillance using meningococcal genome-based Bexsero® Antigen Sequence Typing (BAST).
• Temporal and geographical associations of BASTs and meningococcal clonal complexes.
• BASTs allow real time studies of antigen diversity and estimates of vaccine coverage.

## 1 Introduction

Invasive meningococcal disease (IMD), caused by Gram negative organism *Neisseria meningitidis*, remains an important cause of morbidity and mortality worldwide [1]. In high income regions IMD is normally endemic at low incidence and is associated with serogroups B, C, W and Y, although higher incidence outbreaks occur periodically [2]. Protein-polysaccharide conjugate vaccines have been successfully implemented against the major disease-associated serogroups in high and low income settings, with the exception of serogroup B [3], [4]. The immuno-chemical similarity of the serogroup B capsule and human cell surface polysaccharides, results in poor immune responses and safety concerns [5]. Consequently, development of ‘group B’ vaccines has concentrated on sub-capsular antigens, using either outer membrane vesicles, purified proteins, or both [6]. Bexsero® was licensed in Europe in 2014 and by the end of 2015, two vaccines, Bexsero® [7] and Trumenba® [8], were licensed in the USA. Bexsero®, included in the UK infant immunisation schedule in September 2015 [9], combines the protein antigens factor-H binding protein (fHbp), neisserial adhesion A (NadA), neisserial heparin-binding antigen (NHBA) and PorA with an outer membrane vesicle from the MeNZB™ vaccine [7], [10].

Molecular typing approaches are important for IMD diagnosis in the absence of a confirmed culture [11], [12]; as over half of IMD cases are diagnosed solely by PCR in the UK [13]. Nucleotide sequence-based typing of meningococci has many applications [14], including post-vaccination surveillance, where multilocus sequence typing (MLST) established that meningococcal C conjugate vaccine significantly reduced carriage of the outbreak strain in the UK population [15]. The application of rapid, cost-effective bacterial whole-genome sequencing (WGS), achieved with the Meningitis Research Foundation Meningococcus Genome Library (MRF-MGL), enables high resolution characterisation of clinical isolates at >95% of loci simultaneously [16]. Analysis of MRF-MGL data from 2010/11 onwards, publicly accessible on the PubMLST database [17], made a major contribution to UK vaccination policy, by determining that increases in serogroup W IMD in the UK were due to clonal expansion of an aggressive strain first reported in South America [18], [19].

WGS for disease-causing meningococci in Great Britain and Ireland provides detailed information on vaccine antigen sequence variants and enables inference of vaccine coverage. However, WGS does not identify the extent of immunological cross-reactivity of antigens, especially those with similar but distinct peptide sequences. Therefore, enhanced post-implementation surveillance of Bexsero® currently employs the Meningococcal Antigen Typing System (MATS) assay, developed to predict strain coverage of Bexsero® [20]. Based on serogroup B IMD isolates, strain coverage of 73% in England and Wales was predicted [21]. Here, we characterised the diversity and structure among disease-causing *N. meningitidis* isolates from the UK and Ireland over four years (2010/14), defining a reproducible method for typing Bexsero® vaccine antigens. As a demonstration of the utility of WGS, we developed a model for predicting the likely MATS result from genotype.

## 2 Materials and methods

### 2.1 Genome collections

A total of 2016 genomes were analysed, representing all culture-confirmed cases of IMD for epidemiological years 2010/11 to 2013/14 from Great Britain and Ireland: England (*n* = 1602); Wales (*n* = 120); Scotland (*n* = 114); Northern Ireland (*n* = 47); and Republic of Ireland (*n* = 133) the latter two grouped as ‘Ireland’ for analysis. There were 19 non-groupable isolates, the remainder were serogroups *A* (*n* = 1), *B* (*n* = 1393), *C* (*n* = 88), *E* (*n* = 5), *W*/*Y* (*n* = 4), *W* (*n* = 202), *X* (*n* = 2), *Y* (*n* = 301), and *Z* (*n* = 1). The genomes were hosted on PubMLST *Neisseria* public database (http://pubmlst.org/neisseria/). Embedded tools within PubMLST were used to analyse the presence of Bexsero® vaccine antigens (fHbp, NadA, NHBA and PorA), diversity, association with clonal complex (cc), geographical and temporal spread.

### 2.2 Implementation and curation of Bexsero® Antigen Sequence Type (BAST) scheme

A curated sequence type scheme for Bexsero® antigens was established within PubMLST.org/neisseria database to provide a robust, objective method of comparing the vaccine antigens. This enables easy comparison among datasets collected in different temporal or geographical regions [22]. Previously established nomenclatures for each component were used, with every unique peptide sequence for each Bexsero® antigen (fHbp, NHBA, NadA, PorA-VR1 and PorA-VR2) [23], [24], [25] assigned a unique identification number. Although not included in the MATS assay, PorA-VR1 was included in the BAST scheme, for additional discrimination among vaccine antigens. Each unique combination of these peptide sequences identifiers in an isolate was assigned an arbitrary number (Bexsero® Antigen Sequence Type or BAST) in order of discovery, as sequence types (STs) are assigned in MLST [26]. Assigning peptide sequence variant numbers was complicated where the gene sequence encoding an antigen was absent or did not encode an expressed protein. In the absence of protein expression or functional studies for these loci in all isolates, the most biologically plausible interpretation of sequence data guided the nomenclature. The allele designation 0 (null) was used where absence of the locus encoding a protein in an isolate was confirmed. In those cases where an indel mutation in the *nadA* gene resulted in the gene being phase variable ‘off’, the potential NadA peptide identifier for phase variable ‘on’ was used. For genomes with frameshift mutations in fHbp, NadA, or NHBA that rendered the resultant protein truncated, peptide designation 0 (null) was assigned.

### 2.3 Analysis of antigen diversity and recombination

Simpson’s index of diversity (*D*) assessed the diversity of each protein, ranging from zero to one, with values nearer one indicating greater diversity [27], [28]. Cramer’s V coefficient measured the association of vaccine antigens and BASTs with cc and was calculated using the ‘cramersV’ function in the ‘lsr’ package in R 3.1.1. [29]. Rarefaction curves of BAST and peptide types were produced using the ‘rarefaction’ function in the ‘vegan’ package in R. A rarefaction curve is created by repeatedly re-sampling at random from a collection of *N* individuals and subsequently plotting the average number of species/types represented by *N* individuals. Analysis of recombination in the Bexsero® vaccine antigen genes was carried out using the ClonalFrameML program [30]. ClonalFrameML uses a maximum likelihood approach for phylogenetic reconstruction while taking into account recombination.

### 2.4 Analysis of population structure

The index of association (*I_A_*) determined the level of linkage equilibrium, the random association of alleles at various loci, in sequence data [31]. The ‘standardised index of association’, *I^S^_A_*, detected linkage disequilibrium among the BAST loci using the START2 program 0.9.0 beta [32], [33]. The *f*^∗^ metric measured the amount of non-overlapping structure in the combinations of antigens [34]. A script written and executed in R calculated the *f*^∗^ metric.

Wrights fixation index, *F*_ST_, investigated geographic and temporal structuring. The *F*_ST_ value ranged from zero (no differentiation among groups, indicative of gene flow), to one (complete differentiation and presence of structure in the population). Significant genetic differentiation among groups of isolates (years/regions) and the contribution of geotemporal factors were assessed by AMOVA [35]. *F*_ST_ and AMOVA were calculated with Arlequin version 3.1 [36]. The chi squared test and chi squared test for trend detected changes in BAST over time and calculated with ‘prop.test’ and ‘prop.trend.test’ functions respectively in the ‘stats’ package in R version 3.2.0.

### 2.5 Genotype-phenotype associations

The BAST scheme is a stable nomenclature that acts as a reference which can be correlated with functional assays, for example MATS, which is based on comparison of isolates to reference strains, not individual serum bactericidal assays [20]. We modelled a genotype-phenotype association using 508 invasive serogroup B disease isolates from England and Wales from 2007/08 with previously published genomic and MATS data [21]. The pilot studies using BAST genotype alone were not statistically valid in view of the small sample size (*n* = 508) and high dimensionality of the BAST variable alone (217 levels). Instead, the seven MLST loci (due to associations between cc and BAST) were used with MATS coverage outcome, based on the presence of at least one antigen whose relative potency was greater than positive bactericidal threshold (NadA 0.009, fHbp 0.021, NHBA 0.294) or presence of PorA-VR2:4 or both [21]. A Projection Pursuit Regression (PPR) model was employed to address the high dimensionality in explanatory variables. This was fitted to the data based on overall coverage outcomes of 0 (not covered) or 1 (covered), using RStudio Version 0.99.893. To internally validate the PPR model, 10% of data points were randomly removed and their coverage score predicted, with 80% accuracy for correct predictions. The PPR model was used to predict the coverage of STs in the 2007/08 dataset and these were applied to serogroup B 2010/14 isolates (*n* = 1393).

## 3 Results and discussion

### 3.1 BASTs identified in IMD isolates from Great Britain and Ireland

**Fig. 1:**
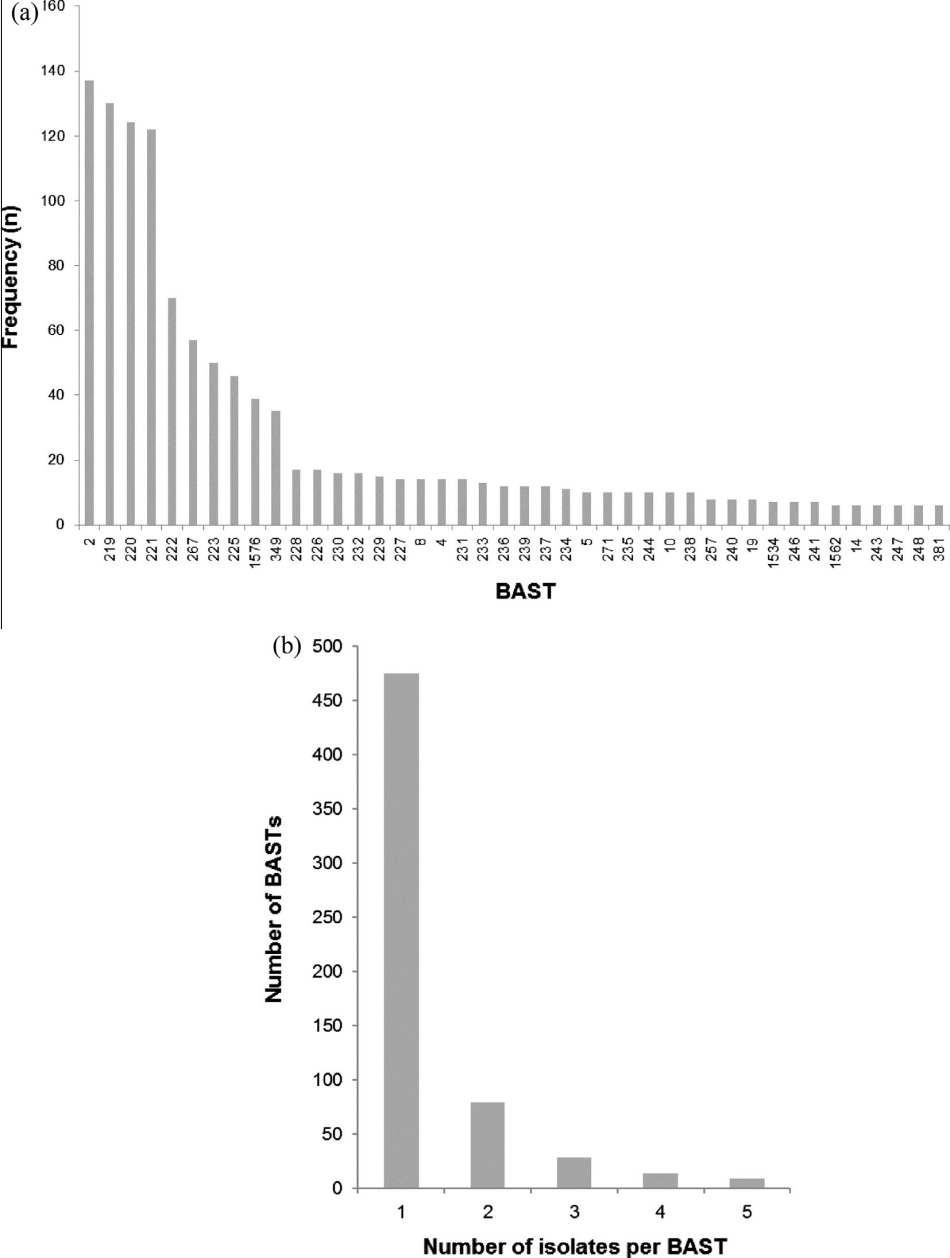
Frequency distribution of Bexsero® Antigen Sequence Types (BAST) in an invasive meningococcal disease collection (*n* = 2016) from England, Wales, Scotland, Northern Ireland and the Republic of Ireland, epidemiological years 2010/11 to 2013/14. (a) Isolates for which the BAST frequency was greater than 5; the *x*-axis indicates the BAST, each numbered BAST-2, BAST-219, etc. Each BAST is a unique combination of peptide sequences of the four Bexsero® vaccine antigens: factor H-binding protein (fHbp); Neisseria Heparin Binding Antigen (NHBA); neisseria adhesion protein (NadA) and outer membrane Porin A (PorA). The *y*-axis is the frequency of each BAST. (b) Isolates for which the BAST frequency was five or fewer. The *x*-axis is the number of isolates and the *y*-axis the frequency of BASTs. Thus, for example, 475 distinct BASTs were represented by single isolates.

Complete BASTs were assigned to 1966 of the 2016 isolates (97.5%) with only 50 (2.5%) isolates with incomplete BASTs. Within the 1966 isolates, 647 BASTs were identified, the nine most frequent accounted for 775 (39.4%) isolates (Fig. 1). A total of 475 BASTs were each represented by single isolates. The most frequent BAST was BAST-2 (fHbp 22; NHBA 29; NadA 6; PorA-VR 1:5; VR 2:2), which accounted for 137 (7.0%) isolates. Each of the vaccine components exhibited peptide sequence variation, with fHbp and NHBA being the two most diverse antigens (Table 1). These data demonstrate that, although there is high diversity in these antigens and therefore many BASTs, a limited number of BASTs occur at high frequency (Fig. S1). Taking into account secular changes, this indicates that a relatively small number of Bexsero® vaccine antigens could achieve high coverage of IMD isolates. There were no occurrences of BAST-1, an exact match to the vaccine antigens [7].

### 3.2 Associations of BAST components with clonal complex

**Fig. 2:**
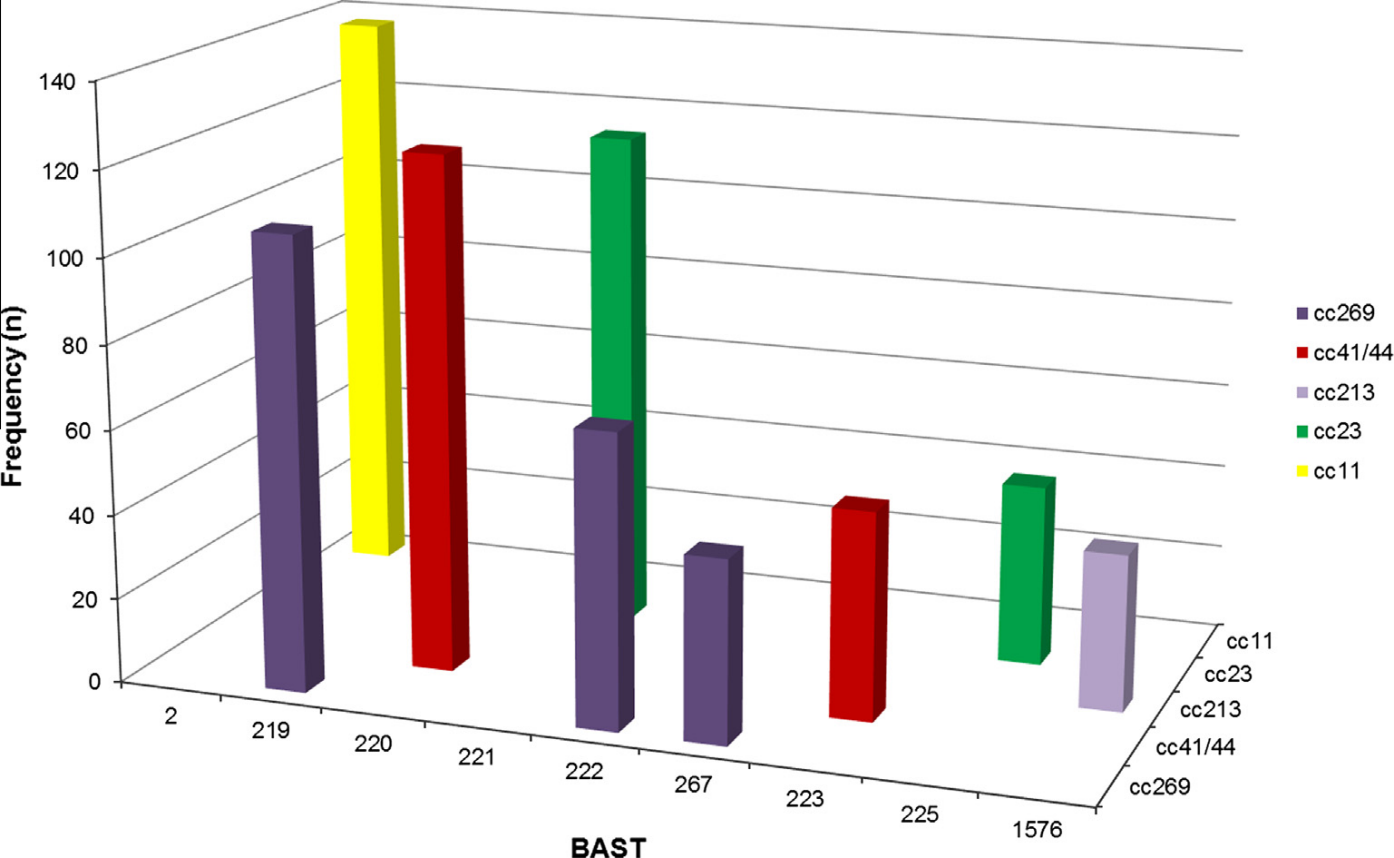
Frequency distribution of Bexsero® Antigen Sequence Typing (BAST) by clonal complex for the nine most frequently occurring BASTs (*n* = 775/2016, 39.4%) in invasive meningococcal disease isolates in the epidemiological years 2010/11 to 2013/14. BASTs shown on the *x* axis are structured by clonal complex (*z* axis) for a proportion of frequently occurring dominant clones circulating in Great Britain and Ireland from 2010/14, for example BAST-2 is only found in isolates of clonal complex 11. Clonal complex 269 has three predominant BASTs, 219, 222 and 267 not found in other clonal complexes.

There were strong non-overlapping associations between BAST and cc as determined by MLST [26] (Figs. 2 and S2). For example, all isolates with BAST-2 (22; 29; 6; 5; 2) (*n* = 137) and BAST-235 (13; 20; 0; 5–1; 10–8) (*n* = 10) belonged to cc11. The nine most frequently occurring BASTs were associated with five ccs, with each cc exhibiting a major BAST (Fig. 2). The association of individual vaccine antigens and ccs was supported statistically by Cramer’s V coefficient (Table 1), where values approaching one support an association with cc.

Such non-random, non-overlapping associations of cc and antigens can be due to clonal descent or immune selection, as predicted by strain structure models [37], [38]. Meningococcal populations have been shown to have a fundamentally non-clonal population structure, as a consequence of extensive horizontal genetic transfer (HGT) [39]. The effect of recombination events on a population was measured with *I^S^_A_*, [31], with values ranging from zero (non-clonal) to unity (clonal). For the whole isolate collection, *I^S^_A_* was 0.301 consistent with a non-clonal population dominated by a limited number of dominant, hyperinvasive lineages.

The overall *I^S^_A_* value in the dataset remained consistent over the first three years (average = 0.297) but increased to 0.403 in 2013/14, most likely reflecting the increase in W:cc11 associated BAST-2. When this BAST was removed from the collection the *I^S^_A_* value decreased to 0.283. This non-random association also held for all pairwise comparisons of antigens with values ranging from 0.330 for the association of fHbp and PorA-VR2 to 0.735 between fHbp and NadA. The effects of HGT resulting in intergenic recombination was detected in each of the antigen genes by ClonalFrame (Fig. S3(a)–(d)), not discussed further here [30].

The non-overlap in antigen repertoires predicted by strain structure models was assessed with the *f*^∗^ metric [34]. Values towards one indicate a non-overlapping structure representing high cross-immunity amongst antigen variants and immune selection maintaining antigenic combinations [38], while values towards zero indicate non-overlapping antigen repertoires and therefore no cross-immunity amongst antigen variants. Similar to the *I^S^_A_* values, the pairwise *f*^∗^ metric calculations produced values ranging from 0.211 for the fHbp and PorA-VR2 pair to 0.614 for fHbp and NadA. The absence of NadA in 1537/2016 (76.2%) isolates and restriction to a small number of lineages is likely to account for the higher *I^S^_A_* and *f*^∗^ metric values compared to the other antigens comparisons. In addition to being consistent with these antigens contributing to the development of natural immunity to the meningococcus, this structure provides an additional means of assessing potential vaccine coverage. Taken together, the patterns of variants within the BASTs in this collection, the distribution of individual antigen variants, and their relationship to cc were consistent with the behaviour of immunodominant antigens in a recombining population as envisaged in strain structure models, which supports the use of combinations of these antigens in vaccine formulations [34], [39].

### 3.3 Distribution of major BASTs by year and geographical region

**Fig. 3:**
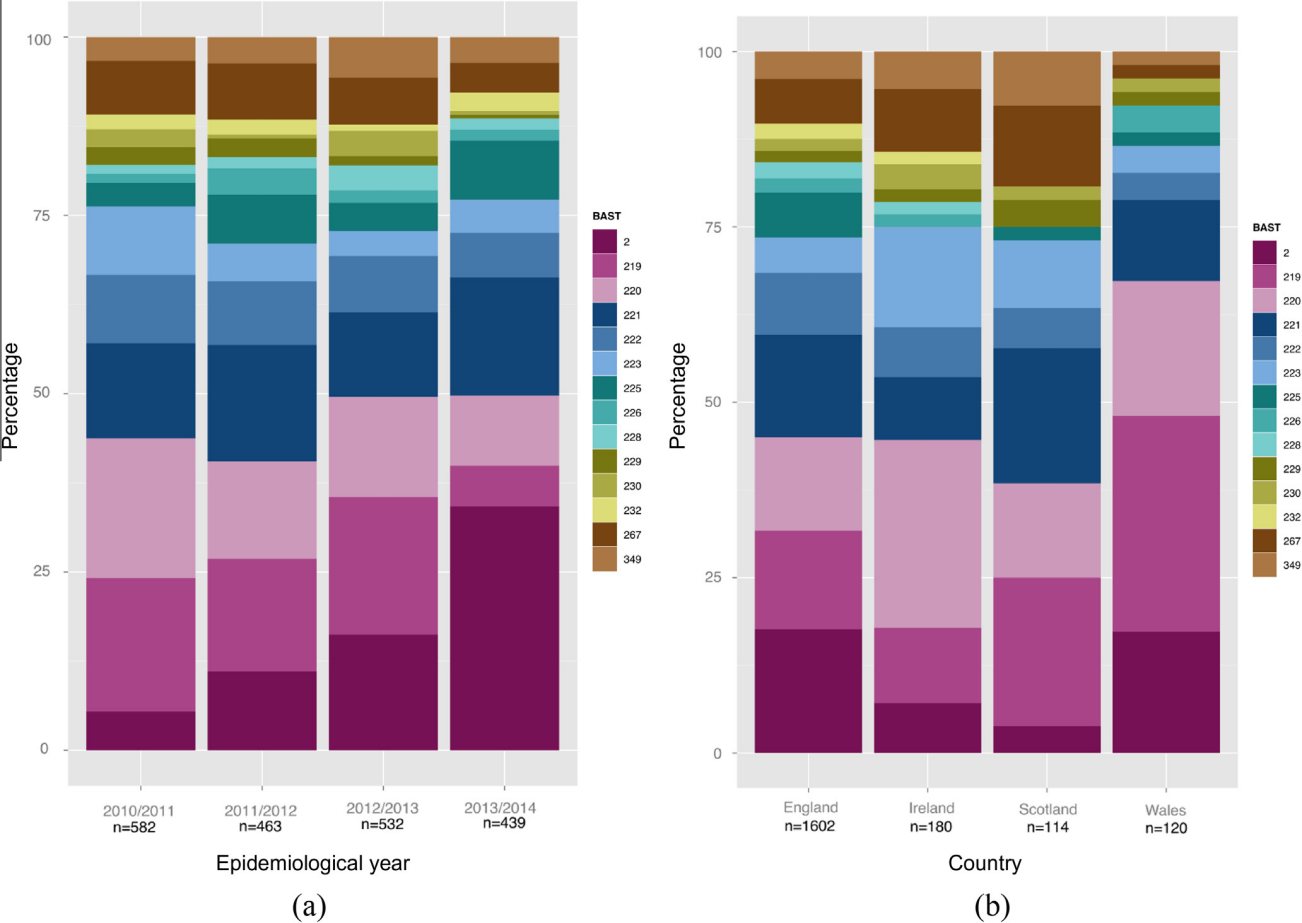
Bexsero® Antigen Sequence Type (BAST) frequency by epidemiological year and by country/region. (a) Comparison of the relative numbers of BASTs represented by more than 15 isolates from 1966 meningococcal invasive disease isolates (Great Britain and Ireland) during each of the four epidemiological years 2010/11 (*n* = 569), 2011/12 (*n* = 449), 2012/13 (*n* = 526) and 2013/14 (*n* = 422). (b) Percentage of BASTs represented by more than 15 isolates from 1966 invasive meningococcal diseases isolates from England (*n* = 1566); Ireland (Northern Ireland and the Republic of Ireland; *n* = 174); Scotland (*n* = 114) and Wales (*n* = 112). Distinct BASTs are colour coded (see key to right of main figure; not related to colour coding used in Fig. 2).

There was a similar distribution of the major BASTs for each epidemiological year (Fig. 3(a)), with the exception of a significant increase related to BAST-2 (cc11) (*χ*^2^ test for trend; *p* < 0.005) which increased in proportion from 2.3% (*n* = 13/569) in 2010/11 to 15.6% (*n* = 66/422) in 2013/14. In the UK, these meningococci caused concern due to atypical and severe clinical presentation [40] and genomic analyses demonstrated their close relationship to South American W:cc11 isolates [18], leading to the implementation of vaccines including conjugate serogroup W polysaccharides. Other notable changes in the nine predominant BASTs were decreases in the two ccs to which most serogroup B strains belong, from 2010/11 to 2013/14. These were cc269 and associated BAST-219 which declined from 7.9% (*n* = 45/569) to 2.6% (*n* = 11/422) (*χ*^2^ test; *p* < 0.005) and cc41/44 and associated BAST-220 which declined from 8.3% (*n* = 47/569) to 4.5% (*n* = 19/422) (*χ*^2^ test; *p* < 0.05).

There was variation in the geographical distribution of BASTs (Fig. 3(b)). For example, at the time of sampling BAST-2 was more prevalent in England (122/1566, 7.8%) and Wales (9/114, 7.9%) than in either Scotland (2/114, 1.8%) or Ireland (4/174, 2.3%). BAST-219 ranged in prevalence from 3.4% of isolates in Ireland to 14.0% in Wales. The eighth most common type in the whole dataset, BAST-225, associated with cc23, was not found in Irish disease isolates. Pairwise comparisons of the four regions using *F*_ST_ values obtained from BASTs were different from the null hypothesis, indicating a significant (*p* < 0.05) subdivision among all regions. It is well-known that the major hyperinvasive ccs are present worldwide and are stable over time [41], [42] but that their prevalence varies among countries [43], [44]. These data are consistent with a generally stable population of invasive meningococci, that varies over time, with occasional large changes in disease incidence caused by the introduction of a novel clone.

### 3.4 Genotypic matches to the Bexsero® vaccine antigens

**Fig. 4:**
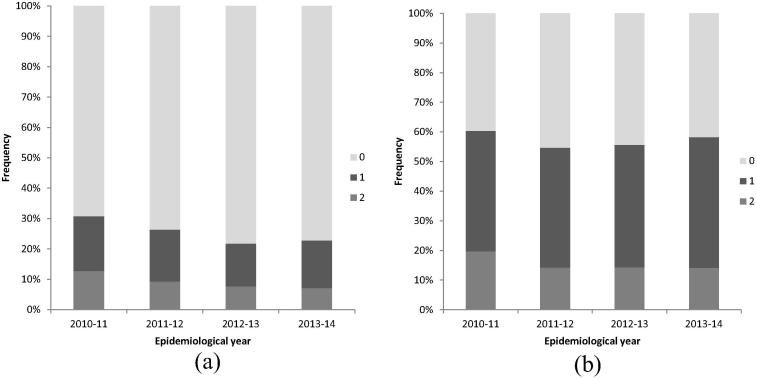
Bexsero® Antigen Sequence Type (BAST) matches to 2016 invasive meningococcal disease isolates in the epidemiological years 2010/11 to 2013/14. (a) All isolates with exact BAST-1 matches shown by percentage of Bexsero® antigen sequence types (BASTs) matching BAST-1 (the antigen profile in the Bexsero® vaccine) at up to four loci (fHbp 1, NHBA 2, NadA 8, PorA VR2 4) by epidemiological years 2010/11 to 2013/14. (b) All isolates with BAST matches to any antigenic variants considered to be cross-reactive (fHbp variant 1 peptides: 1.1, 1.4, 1.13, 1.14, 1.15, 1.37, 1.232, any NadA peptide variants: NadA-1: 1, 100, 137, 141, NadA-2/3: 2, 3, 4, 6, 7, 8, 121, 127, 130, 131, NadA-4/5: 21, PorA VR1 7.2), shown by percentage of isolates BASTs matching up to two loci by epidemiological years 2010/11 to 2013/14 (there was only one isolate in 2013/14 with three matches to cross reactive loci).

Isolates were compared at four loci within the vaccine BAST-1, fhbp:1, NHBA:2, NadA:8 and PorA-VR2:4, as PorA-VR2 is included in the MATS assay where PorA-VR1 is not [20]. No isolates in the dataset had an exact match to the vaccine BAST-1. There were two peptide matches in 74/582 (12.7%) isolates from 2010/11 which reduced to 31/439 (7.1%) in 2013/14 (Fig. 4(a)); however, it has been proposed that immunogenicity to only one of the four vaccine targets is sufficient to protect against disease [45]. Over the four years, the proportion of isolates with at least one vaccine antigen matching to BAST-1 decreased from 179/582 (30.8%) to 100/439 (22.8%). Predictions based on antigen peptide sequence alone therefore indicated that a large proportion of disease-causing isolates would not be covered by the vaccine; however, this does not take into account the potential contribution to protection afforded by antigen cross-reactivity.

To account for this, MATS vaccine antigen coverage, which acts as a proxy based on recombinant antigens, was used to identify cross-reactive variants. The fHbp variants considered cross-reactive were: 1.1; 1.4; 1.13; 1.14; 1.15; 1.37; 1.232 [21]. As there was no correlation between nucleotide sequence variants and relative potency of NadA, presence of any NadA variant was considered cross-reactive for the purposes of this estimate. The presence of PorA-VR1:7-2 was included due to the potential immunogenicity of this antigen. Due to lack of phenotypic data regarding the immunogenicity of NHBA variants, this antigen was excluded from this analysis. The proportion of isolates possessing at least one cross-reactive vaccine antigen ranged from 58.3% to 60.3% (Fig. 4(b)). We conclude that the remaining 39.7–41.7% would likely be dependent on protection through antibodies to NHBA, for which there is a lack of published information.

Vaccine coverage in England and Wales in 2007/2008 was estimated using the MATS assay [21]. This identified 73% (95% CI 57–87%) of isolates that possessed at least one vaccine antigen exceeding the relative potency threshold or presence of PorA-VR P1.4 or both. Vogel et al., deem this a conservative estimate, owing to limitations imposed by using pooled toddler sera as opposed to adult or adolescent sera, and lack of consideration of the co-operation and interactions of minor antigens or those below the positive bactericidal threshold [21]. As the isolates used in this analysis of BASTs were collected between three and seven years after the time period for which coverage of 73% was estimated, secular changes in the bacterial population may contribute to these differences, but it may also be attributable to differences in the approaches used.

### 3.5 Genotype-phenotype associations

Using increasingly available WGS, BAST provides a first step in identifying potential vaccine coverage. As this metric does not take into account cross-protection and phenotypic expression, it is important to correlate BAST analysis with functional assays, such as MATS, and ultimately with disease incidence in immunised and unimmunised individuals. The latter are more difficult to quantify robustly, therefore assumptions were made, but these associations were based on the stable BAST nomenclature. To demonstrate the potential utility of linking genotype and phenotype, published data from 2007/08 [21] was used to fit a PPR model using the seven MLST loci (given the strong associations between cc and BAST) and vaccine coverage outcomes (0 = not covered, 1 = covered). The predicted coverage for each ST in 2007/08 was applied to serogroup B 2010/14 isolates for which an ST was available (*n* = 1393). There were 887/1393 (63.7%) isolates for which coverage could be predicted, the remaining 506 had STs not present in 2007/08. For 2010/14, the predicted coverage for individual epidemiological years ranged from 63.0% to 73.0%. The mean coverage estimate for 2010/14 was 66.1% (95% CI 61.5–70.7%). The proportion of isolates for which prediction was possible diminished over the four years; 68.3% in 2010/11 to 51.6% in 2013/14. In this study, the limitation of using BAST as opposed to ST, was the variation in number of unique BASTs, 217 in 2007/08 (0.43 BASTs/isolate) which increased to 647 in 2010/14 (0.33 BASTs/isolate). As more MATS data becomes available for WGS isolates in larger datasets, this statistical modelling may become possible. The difference in coverage for a given ST, as indicated by the MATS assay, where the host response is assumed to be constant, presumably reflects inter-strain variation in antigen expression. As Bexsero® surveillance continues, this model therefore provides predictions controlling for the host immune responses, a potentially added complexity encountered when analysing the impact of isolates causing disease in vaccinated individuals. It also allows estimations of vaccine coverage for isolates where STs are already available in the absence of WGS. For example, the EUMenNet collection (2413 isolates across 17 countries from 2000/2002) [44] had 1240 isolates for which coverage could be predicted, and was estimated at 88.8% (95% CI 85.8–91.8%).

## 4 Conclusions

In this large and representative collection of IMD isolates from Great Britain and Ireland between 2010/14, we have shown diversity of BASTs, their correlation with genetic structure, and variability over time and by geographic region. This analysis demonstrates a means of achieving rapid and scalable surveillance of isolates from IMD for Bexsero® vaccine antigens. The number of BASTs not previously present in these regions [21], demonstrated how the diversity of IMD isolates alters over time; however, a number of BASTs were relatively stable, W:cc11 associated BAST-2 was an exception. Based on these analyses, mean estimates of vaccine coverage were 66.1% (95% CI 61.5–70.7%), but further disease surveillance occurring in newly vaccinated infants will inform about cross-reactivity and coverage. Finally, BAST provides a tool for studying the distribution of vaccine antigens present in the stable and persistent ccs in any population, presenting the opportunity for reformulation of the current Bexsero® vaccine if and when required.

## Funding

The work was supported by Meningitis Research Foundation. We made use of the Meningitis Research Foundation Meningococcus Genome Library developed by Public Health England, the Wellcome Trust Sanger Institute, and the University of Oxford. The Meningitis Research Foundation Meningococcus Genome Library is part of the *Neisseria* Sequence Typing database website developed by Keith Jolley and Martin Maiden and hosted by the University of Oxford and supported by the Wellcome Trust (Grant 104992). CMCR was supported by Wellcome Trust (Grant 109031/Z15/Z). MCJM had been supported by the Wellcome Trust (Grant 087622).

## Conflict of interest statement

CMCR, CB, RB, AS and RC declare no competing interests. MCJM has received grants and personal fees from Novartis outside the submitted work. RM is a scientific adviser to GSK and a member of the Scientific Advisory Board of LimmaTech Biologics AG, Zurich.

## Figures and Tables

**Table 1:** Bexsero® antigen diversity and associations in meningococcal genome collection from Great Britain and Ireland between epidemiological years 2010/11 to 2013/14.

Peptide	No. of variants	Index of diversity (*D*)	Cramer’s V (association with cc)	*f*^∗^ metric				Standardised index of association			
(95% CIs)	fHbp	NadA	NHBA	PorA-VR1	fHbp	NadA	NHBA	PorA-VR1
fHbp	165	0.920 (0.915–0.925)	0.651								
NadA	17	0.688 (0.643–0.732)	0.489	0.614				0.735			
NHBA	140	0.914 (0.909–0.919)	0.548	0.321	0.521			0.506	0.647		
PorA-VR1	39	0.871 (0.864–0.878)	0.716	0.229	0.633	0.255		0.330	0.648	0.380	
PorA-VR2	87	0.919 (0.915–0.923)	0.497	0.211	0.492	0.273	0.471	0.345	0.635	0.442	0.597
BAST	647	0.974 (0.971–0.977)	0.946								
