# Supplementary material for: Distribution of Bexsero® Antigen Sequence Types (BASTs) in invasive meningococcal disease isolates: Implications for immunisation
Source: Vaccine. 2016 Sep 7;34(39):4690–7. doi: 10.1016/j.vaccine.2016.08.015 (PMC5012890; doi:10.1016/j.vaccine.2016.08.015)
Supplement: Supplementary data 1 [file mmc1.ppt]

## Slide 1
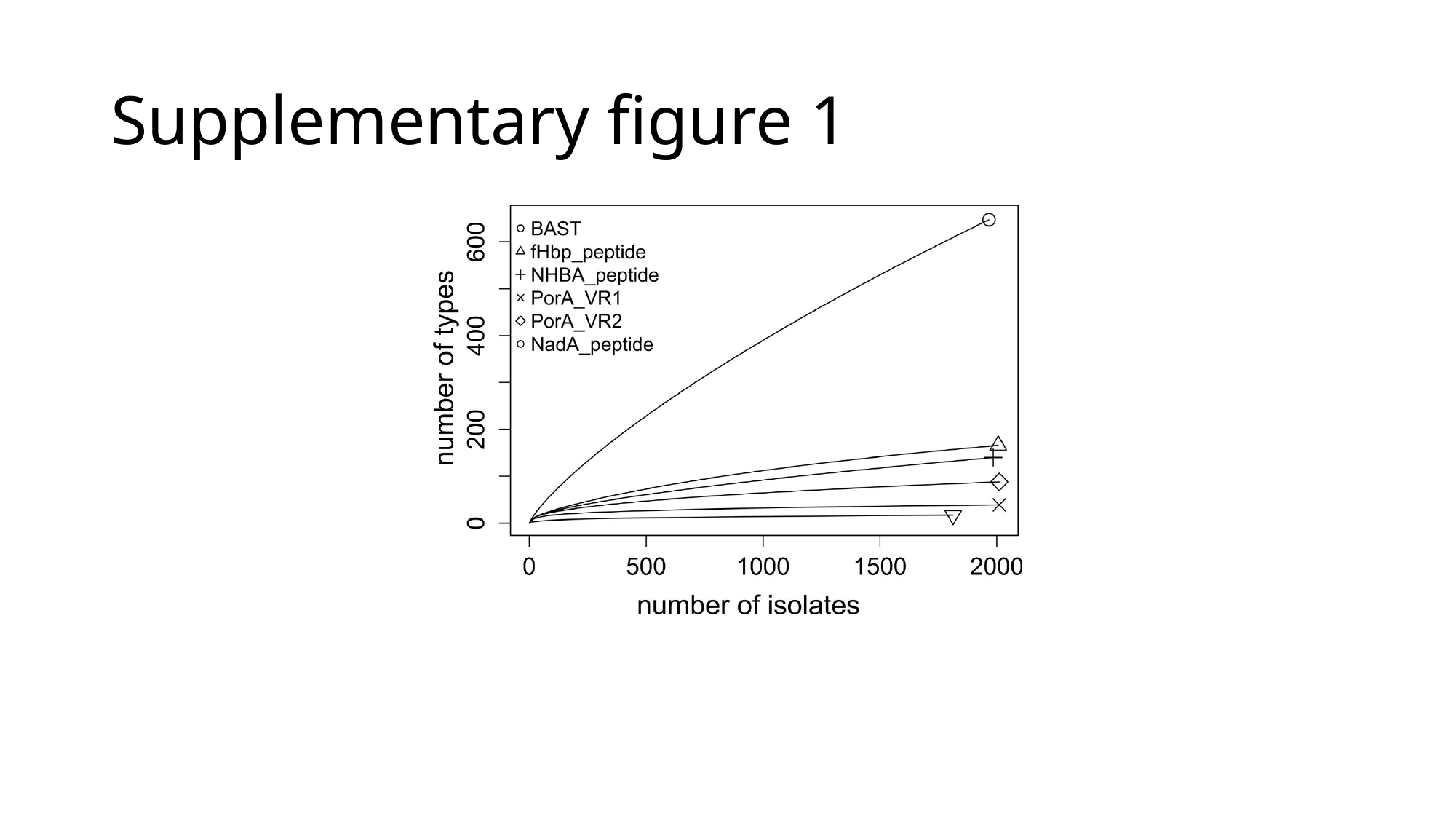

# Supplementary figure 1

## Slide 2
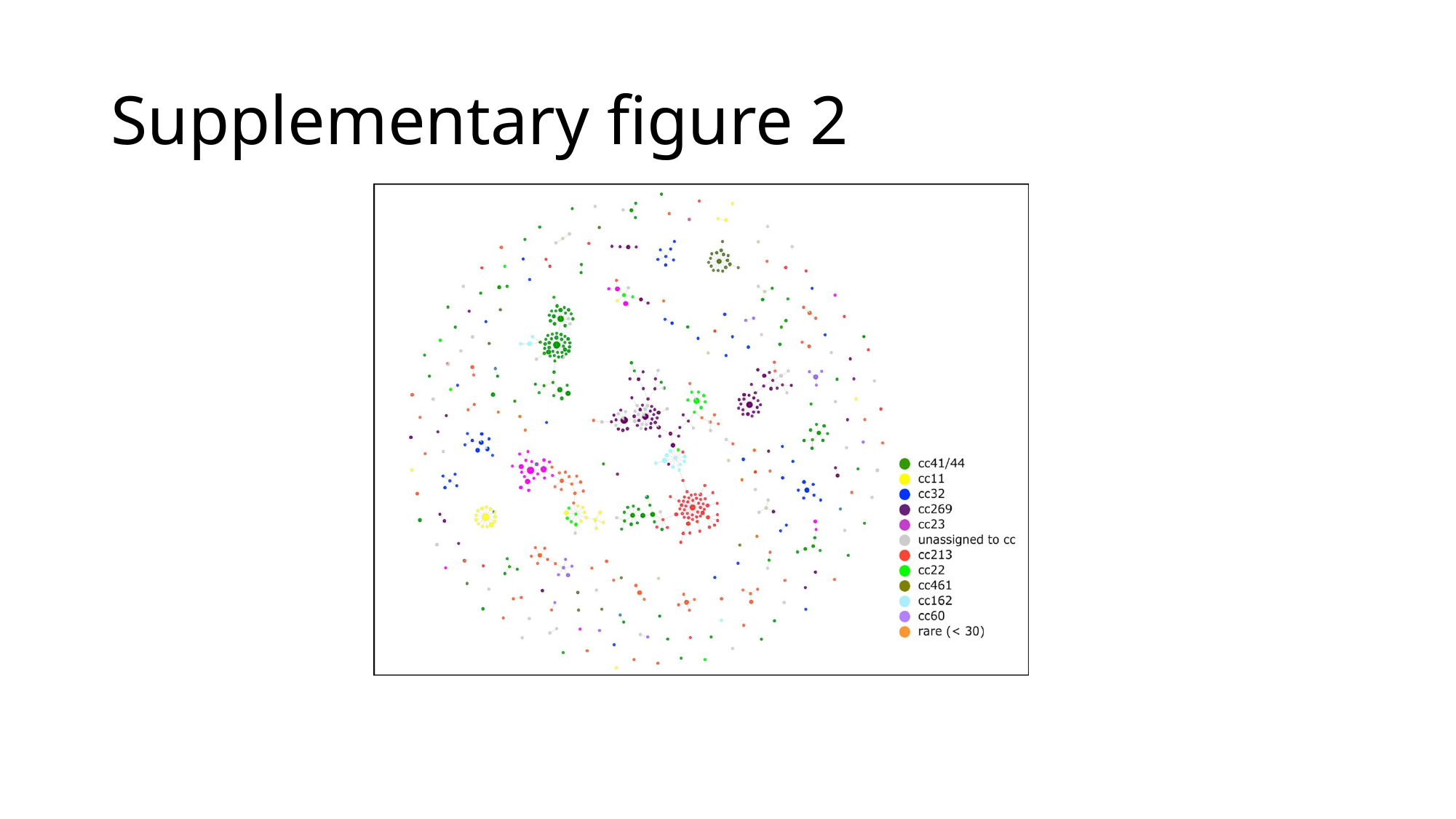

# Supplementary figure 2

## Slide 3
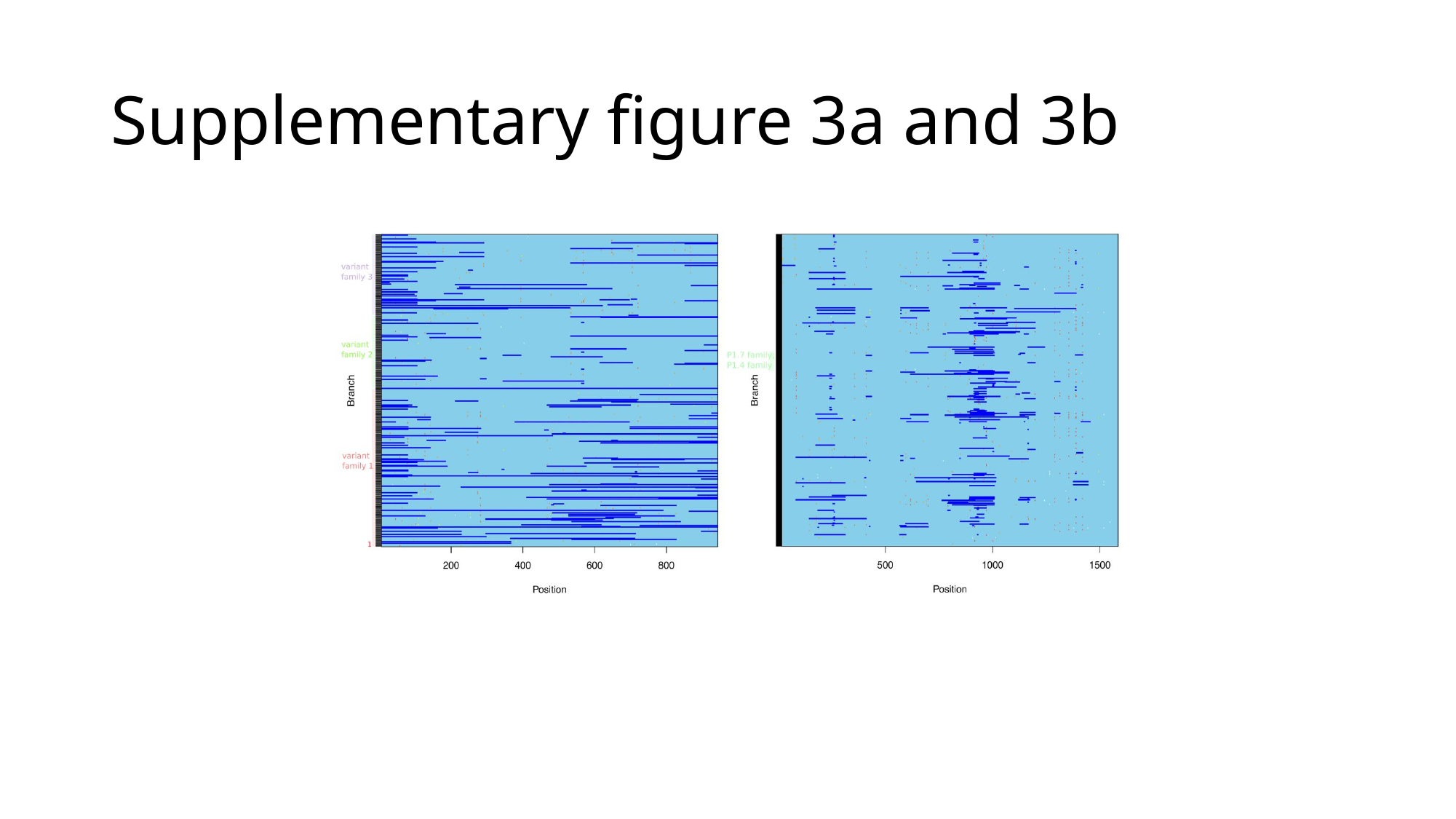

# Supplementary figure 3a and 3b

## Slide 4
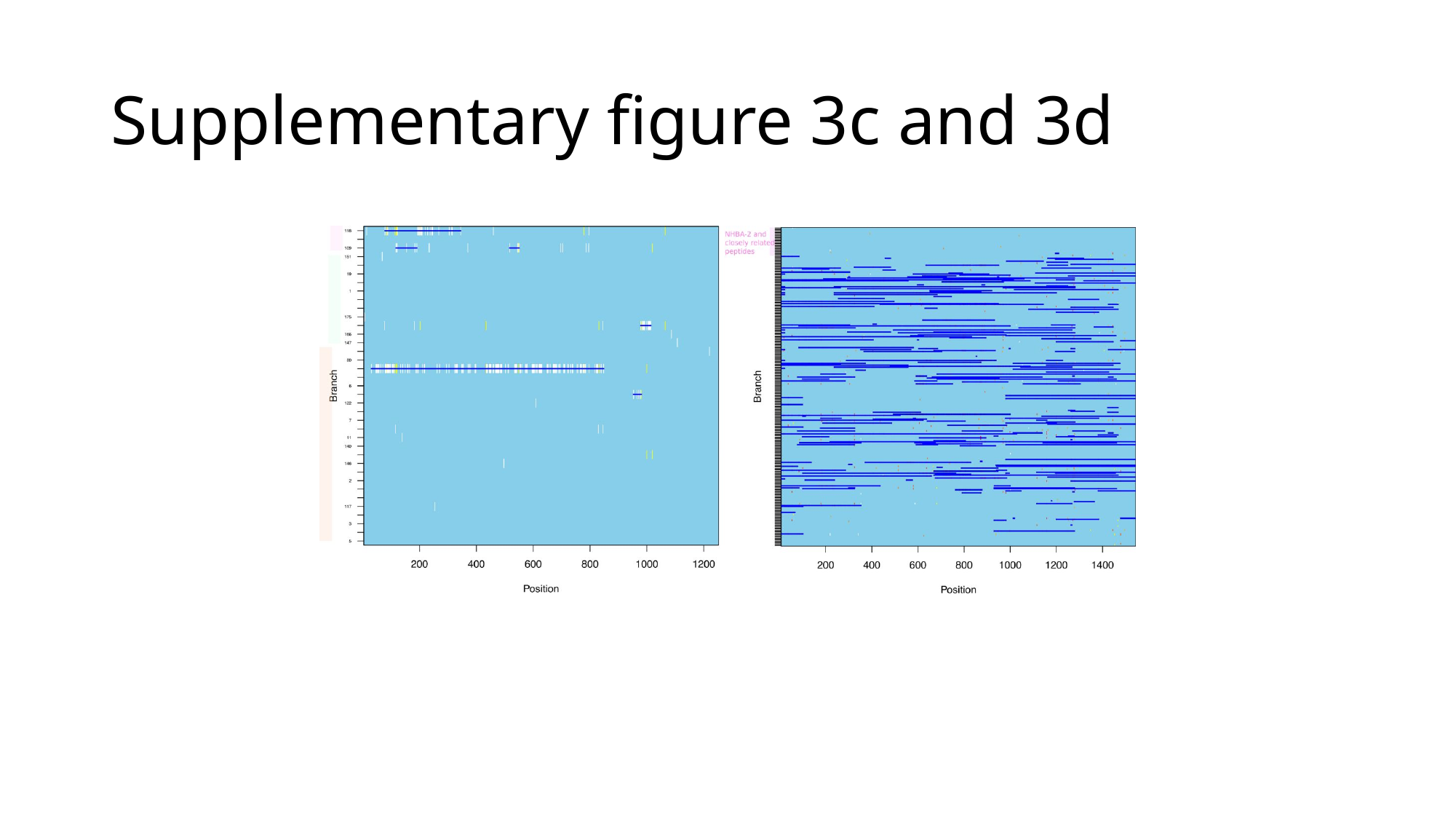

# Supplementary figure 3c and 3d
