# Supplementary material for: Distribution of Bexsero® Antigen Sequence Types (BASTs) in invasive meningococcal disease isolates: Implications for immunisation
Source: Vaccine. 2016 Sep 7;34(39):4690–7. doi: 10.1016/j.vaccine.2016.08.015 (PMC5012890; doi:10.1016/j.vaccine.2016.08.015)
Supplement: Supplementary data 2 [file mmc2.docx]

***Supplementary figures***

**Figure S1: Rarefaction curve of increase in types (BAST, fHbp peptide, NadA peptide, NHBA peptide and PorA type) with number of isolates.**

**Figure S2: Predominant BAST clusters defined using the goeBURST algorithm in PHYLOViZ and coloured by clonal complex.**

Clusters were defined at the single locus variant level (SLV) i.e. varying at one of the five peptide variants NHBA, fHbp, NadA, PorA VR1 or VR2 with colouring for each BAST based on clonal complex association. Each circle represents one of the BASTs found in the dataset and the size of the circle corresponds to the number of isolates with that particular BAST.

**Figure S3: Detection of recombination in Bexsero® antigen alleles; (a) fHbp, (b) porA, (c) nadA, (d) nhba using ClonalFrameML [30]**. ClonalFrameML uses a maximum likelihood approach for phylogenetic reconstruction while taking into account recombination. For any branch of the genealogy and any position along the sequence, inferred recombination is indicated by blue horizontal bars.
